# Successful Use of Argon Plasma Coagulation in the Treatment of Multiple Recurrent Lower Tract Papillomatosis: A Case Report

**DOI:** 10.1002/rcr2.70509

**Published:** 2026-02-16

**Authors:** Samer El Rayess, Ahmad Doklaigah, Wassim Hamadeh, Behnaz Saadieh, Hani Shahin

**Affiliations:** ^1^ Makassed General Hospital Beirut Lebanon; ^2^ Beirut Arab University Beirut Lebanon; ^3^ Lebanese University Faculty of Medical Sciences Beirut Lebanon

**Keywords:** argon plasma coagulation, human papilloma virus (HPV), pneumology, respiratory papillomatosis

## Abstract

Recurrent Respiratory Papillomatosis is a rare, benign papillomatous growth of the bronchial epithelium which occurs in 18 patients in a million. Due to the rareness of the disease, no general treatment consensus exists. Surgical debulking or simple excision via bronchoscopy are the most used therapeutic approaches with adjunct medical therapy, such as intra‐lesional antivirals and interferon therapy, in case of recurring disease. We present the case of a 51‐year‐old man who presented with recurrent respiratory papillomatosis. This case made the approach more challenging as we were dealing with widespread isolated lower respiratory tract involvement (lower trachea and main bronchi) sparing the larynx and vocal cords without evidence of HPV virus infection. A decision was made to intervene with bronchoscopic electro snare and argon plasma coagulation (APC) as few reported cases highlighted the effectiveness of APCs in the treatment of Recurrent Respiratory Papillomatosis with the patients remaining lesion‐free in the 5 months follow up. Moreover, its safety and low risk of perforation/cartilage damage along with its efficiency when it comes to multiple lesions in critical sites established its important role as a therapeutic option.

## Graphical abstract

Recurrent Respiratory Papillomatosis is a rare, benign papillomatous growth of the bronchial epithelium which occurs in 18 patients in a million. Due to the rareness of the disease, no general treatment consensus exists. We present the case of a 51‐year‐old man who presented with recurrent respiratory papillomatosis and was treated with bronchoscopic electro snare and argon plasma coagulation. There was no adverse effect, and the patient was symptom free at the 5 months follow up.

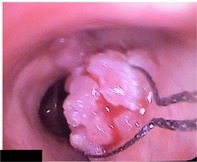

## 1 Introduction

Recurrent respiratory papillomatosis (RRP) is a benign papillomatous growth of the bronchial epithelium, rare in adults, with an incidence of 18 patients in a million [1, 2, 3, 4]. It is thought to be due to Human Papilloma Virus (HPV) infection in about 90% of the cases [1]. Isolated lesions in the bronchial tree are not reported as frequently as isolated upper airway papilloma or concomitant laryngeal involvement with tracheal polyps.

Due to its rarity, and up to this date, no treatment guidelines have been established. Herein, we report the successful use of Argon Plasma Coagulation (APC) in treating a patient with recurrent respiratory papillomatosis.

## 2 Case Report

**FIGURE 1:**
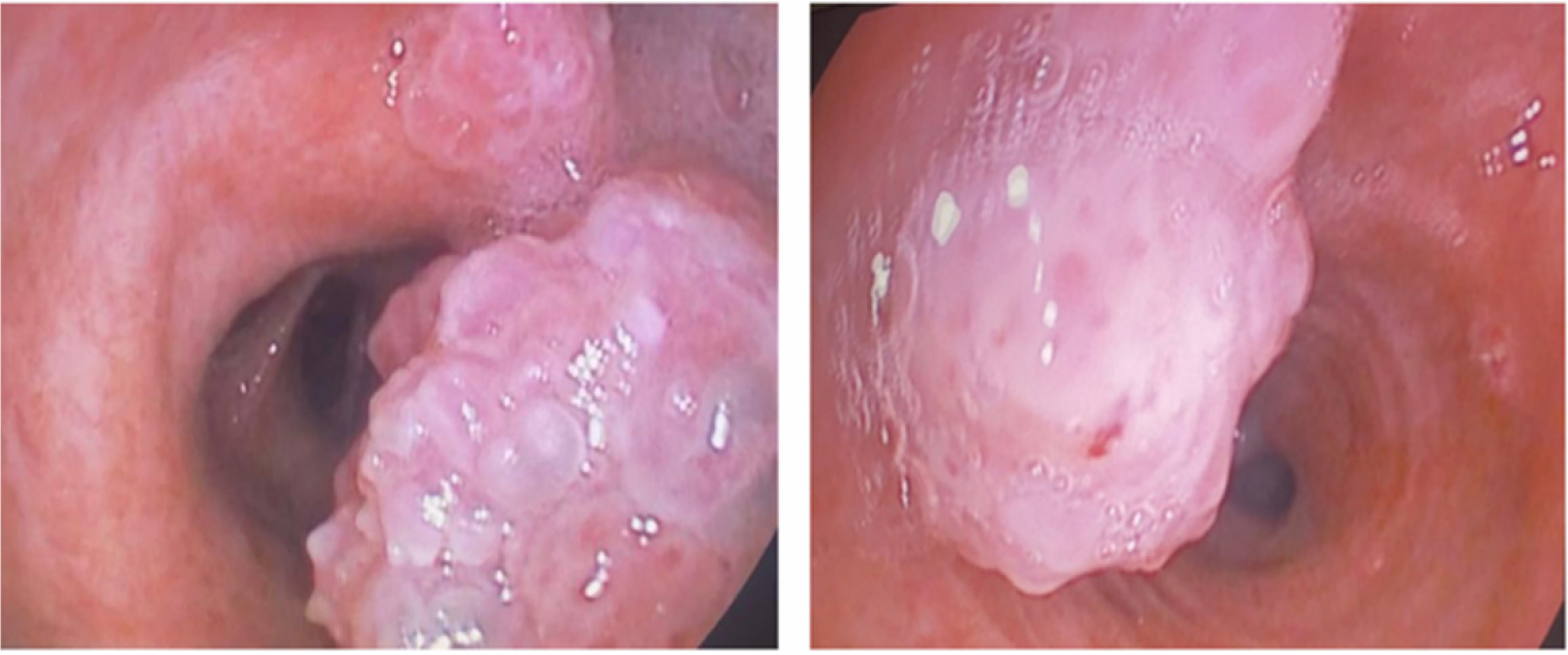
The Intra‐bronchial lesions visualised by flexible bronchoscopy.

**FIGURE 2:**
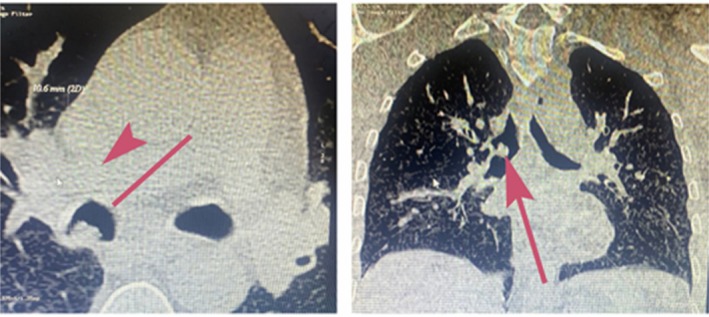
Computed tomography (CT scan) showing a lesion in the right bronchus (red arrow).

**FIGURE 3:**
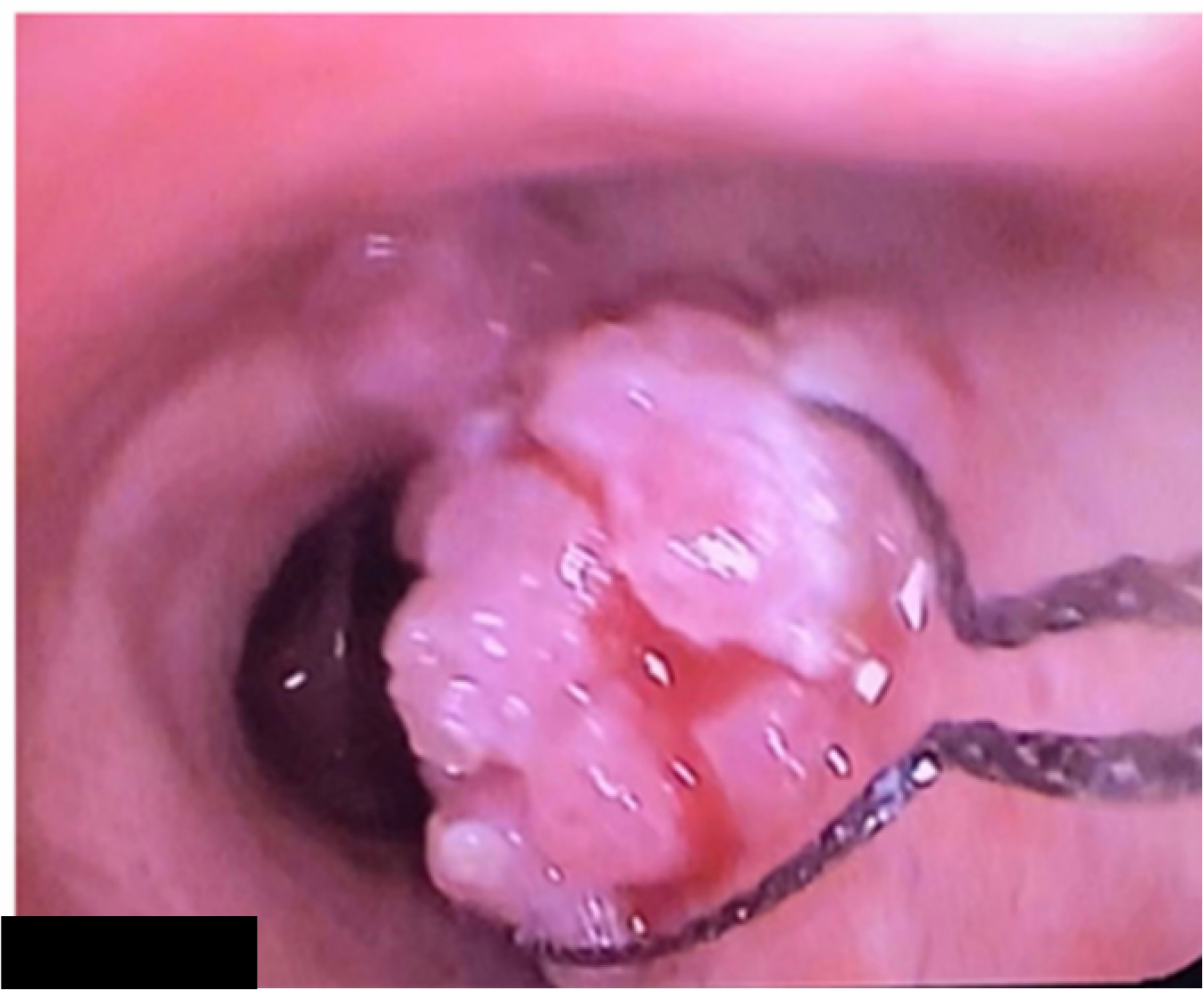
Hot snare around the peduncle.

**FIGURE 4:**
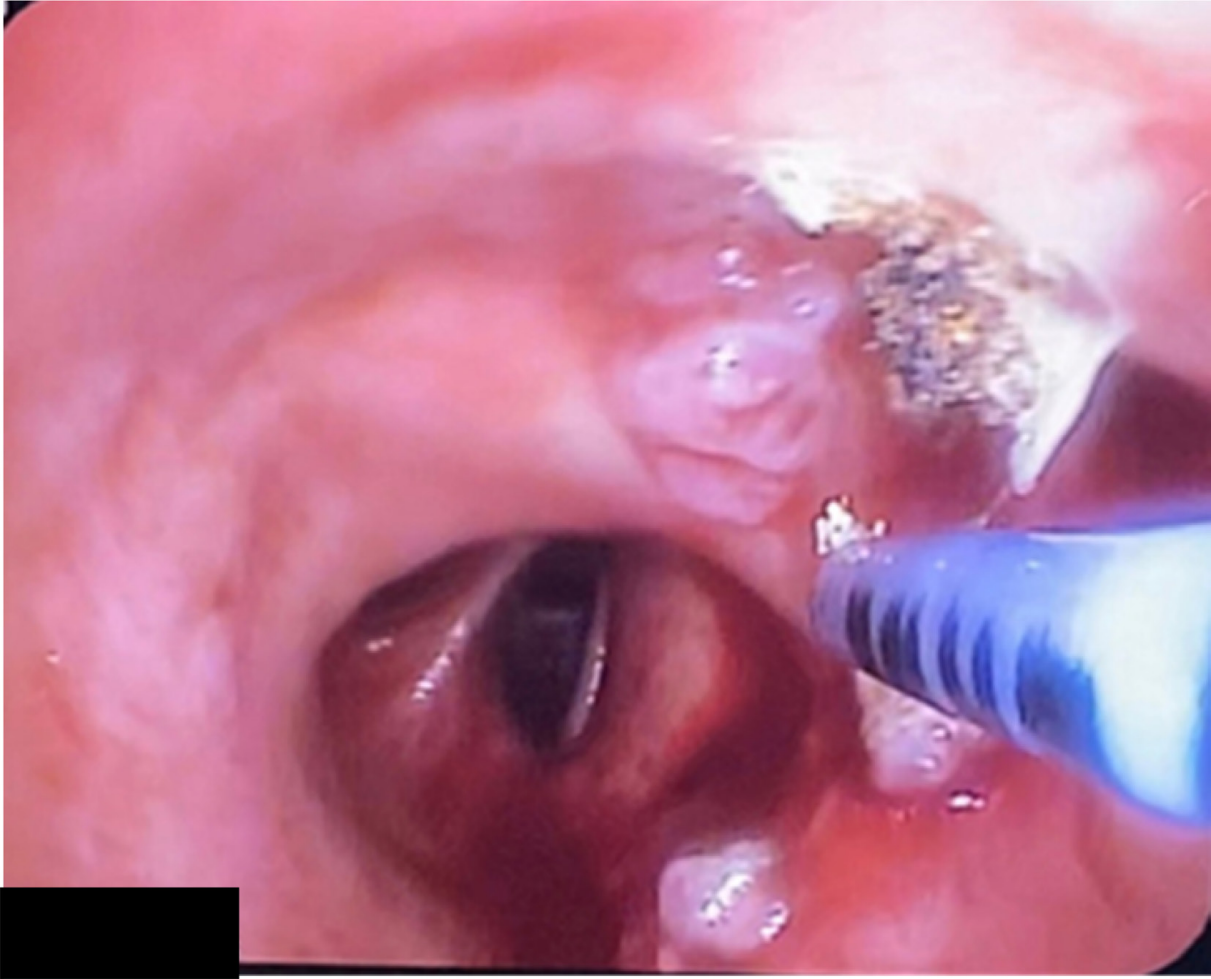
APC probe over the region.

A 51‐year‐old man, complaining of hemoptysis, presented to our clinic. He is a heavy smoker (30 packs year). He didn't report significant weight loss over the past months, anorexia, or night sweats. His past medical and surgical histories were unremarkable. He mentioned that a bronchoscopy was done 1 year ago to investigate the cause of hemoptysis. At that time, multiple biopsies were taken from endobronchial lesions (Figure 1). Pathology revealed squamous papilloma with moderate dysplasia. No further actions were taken at that time. Bronchoscopy repeated after 1 year when he presented to our clinic showed an increase in the number of lesions. Removal of the polyps was done in a two‐step procedure in the Operation Room (Each intervention lasted approximately 45 min and was performed 1 week apart) under general anaesthesia using the APC technique and thermal ablation. Flexible bronchoscopy was used. The polyps were snared around the peduncle and detached from the bronchial wall; then APC was applied in a continuous manner onto the tissue with around 40–50 watts of energy escalating depending on the lesion, until clearance of the airway from any visualised polyps (Figures 2, 3, 4). No adverse effect occurred after 5 months.

## 3 Discussion

RRP is a benign progressive growing tumour which affects mainly upper airways being focal or diffuse, with various sites of involvement, sizes and rate of growth [1]. Thus, it has an unpredictable course and non‐specific symptoms and signs. Despite the benign nature of the tumours, it carries a high morbidity when it comes to malignant transformation in 3%–4% within 10 years of diagnosis, or when it complicates recurrent respiratory infections owing to mechanical obstructions or bronchiectasis and spread throughout the respiratory tract [1]. Though only a few are idiopathic, it is believed that most respiratory papilloma are attributed to HPV, a virus known not only for driving recurrent disease but also for its potential to undergo malignant transformation in a subset of patients. Hence, they tend to occur with bimodal age distribution; in paediatrics age its transmission is via vertical and in adults via reactivation of latent HPV carriers or sexual transmission, as thought to be in our case. So far, there is no conventional treatment for RRP, with debulking or excision via bronchoscopy being the mainstay therapy and diagnostic tool. Adjunct medical options are required in around 20% of the cases when the disease tends to recur rapidly, spread distantly, causing airway obstructive symptoms or when frequent bronchoscopies are required [5]. Aside from the absence of definitive cure of the disease, what made our case more challenging is the unusual widespread of multiple isolated lower respiratory tract involvement (lower trachea and main bronchi) sparing the larynx and vocal cords without evidence of HPV virus infection. Secondly, our patient was already in a relapse with more spread given he had undergone bronchoscopic resection 1 year prior to presentation. This, in turn, rendered him a candidate for the use of adjunct therapies (rapid recurrence with airway compromise) like antiviral and interferon. However, a decision was made to intervene with bronchoscopic electrocauterization and APC due to the unavailability of the adjunctive therapies. This decision was supported firstly in the literature, by a few reported cases emphasising the effectiveness of RRPs treated with APCs and secondly, owing to the wish of our patient who refused to take the quadrivalent vaccine of HPV, which was proven to decrease papilloma growth rate and consequently the number of bronchoscopies. All in all, multiple factors made APC an optimal therapy option for our patient. First, its safety and low risk of perforation/cartilage damage (no contact) along with efficiency when it comes to multiple lesions with critical sites through the bronchi. Second, avoidance of systemic side effects of the anti‐viral and bevacizumab beside the cost effectiveness. Third, APC, when compared to other modalities of bronchoscopy like laser, brachytherapy or cryotherapy, was found to be of the most evidence according to similar published case reports. Finally, APC use in RRP, which was first described in the literature in a few reports, shows promises for a cure, as it has shown success in preventing relapse of the disease [1, 2, 3, 4, 5].

In conclusion, bronchoscopic Argon Plasma Coagulation shows promising success as a treatment option for Reccurent Respiratory papillomatosis, with minimal post‐operative complication and a 5 month symptom‐free period in our patient. Furthermore, when compared to other modalities such as bronchoscopic laser, brachytherapy or cryotherapy, APC was found to be the most effective at reducing the rate of relapse according to similarly published case reports [1, 2, 3, 4, 5].

## Consent

The patient provided written informed consent for his case to be published using the form provided by the journal.

## Conflicts of Interest

The authors declare no conflicts of interest.

## Data Availability

Data sharing not applicable to this article as no datasets were generated or analysed during the current study.
